# Dr. Jesse C. Mosshammer

**Published:** 1908-07

**Authors:** 


Dr. Jesse C. Mosshammer, of Dayton, O., died at his home in that city May 27, 1908, from pleuropneumonia, aged thirty-six years. He graduated at the University of Buffalo, medical department, in 1904. He was ill for about ten days.

